# Editorial: Post-transplant haematological disorders in solid organ transplantation

**DOI:** 10.3389/fimmu.2026.1811503

**Published:** 2026-04-01

**Authors:** Giovanbattista Ippoliti

**Affiliations:** National Transplant Centre- Rome and Policlinico di Monza, Monza, Italy

**Keywords:** clinical presentation, diagnostic procedures, immunosuppression, post transplant hematological disorders, solid organ transplantation (SOT)

When assessing haematological complications following solid organ transplantation (SOT), it is important to consider transplant-specific factors such as timing, immunosuppression intensity, infectious complications, and organ function, all of which significantly influence their onset. Cytopenias, haemophagocytic syndrome, transplant-associated thrombotic microangiopathy, and post-transplant lymphoproliferative disorders commonly occur within the first few months or years after transplantation, typically during periods of more intense immunosuppression, although late manifestations have also been reported. Anaemia and other forms of cytopenia can result from multiple mechanisms, most commonly drug-induced bone marrow suppression related to concomitant immunosuppressive or myelotoxic agents. However, prolonged and intensive immunosuppression increases the risk of infections, neoplasms, and drug-related toxicities, including haematological complications such as anaemia and cytopenias. In these cases, the dosage of immunosuppressive drugs must be adjusted, potentially reducing their effectiveness and increasing the risk of acute rejection and transplant failure.

​Early diagnosis of pancytopenia through bone marrow biopsy is warranted, as it is often the presenting symptom of one or more pathologies following bone marrow transplantation. These complications may have a better prognosis if early intervention is undertaken. Most of the published articles focus on specific topics such as PTLD, cytopenia, anaemia, and so on. These complications have various causes and can result from both immunosuppressive therapy and changes in immune system activity following transplantation.

The clinical presentation of blood disorders is similar to that of non-transplanted individuals; however, the progression, prognosis, and treatment differ due to the anergic state in post-transplant patients. The accepted articles on this topic have explored the subject from a variety of perspectives.

Carlier et al. address this topic by evaluating haematological complications in transplant patients with altered telomere biology. Telomere biology disorders (TBD) are emerging as a critical, yet under-recognised risk factor in solid organ transplantation, transforming what was once considered routine haematological management into a field requiring precise diagnosis and immunosuppression. tailor-made. Defects in the telomere maintenance mechanism lead to premature telomere shortening, resulting in cellular senescence, apoptosis, and organ dysfunction, collectively known as telomere biology disorders. TBDs are recognised as a significant risk for haematological complications associated with immunosuppressive drugs.

The impact of TBD-related haematological complications on the outcome of solid organ transplants is limited to small series of heart and kidney transplants. Conversely, the majority of existing evidence comes from lung transplant cohorts.

Lung transplantation has served as a clinical “sentinel” for TBD, as a significant subset of patients with interstitial lung disease (ILD) carry pathogenic variants in telomere-related genes and exhibit telomere lengths below the first or tenth percentile. In several cohorts of patients with IPF or ILD who carry TERT, TERC, RTEL1, or PARN variants, post-transplant cytopenias are surprisingly common. Anaemia, neutropenia, and thrombocytopenia requiring transfusion occur in the vast majority of cases, often leading to a switch from triple to dual immunosuppressive therapy. In a series of cases, almost all carriers of the variant developed leukopenia and anaemia, and a significant proportion also developed thrombocytopenia. Complications were more common in patients with pre-transplant thrombocytopenia or a history of SMD, highlighting the limited bone marrow reserve typical of TBD.

Similar concerns arise regarding liver transplantation in patients with short telomere syndromes. While positive transplant outcomes have been reported, these cases often involve a history of prior hematopoietic stem cell transplantation, ganciclovir-resistant CMV infection, and a persistent risk of hematological and immune complications.

The pharmacological arsenal used in transplantation adds further myelotoxicity to this already fragile baseline situation. Antimetabolites such as azathioprine and mycophenolate mofetil, calcineurin and mTOR inhibitors, and prolonged exposure to corticosteroids all carry dose-dependent and duration-dependent risks of cytopenia and impaired immune competence. Anti-infective prophylaxis, particularly valganciclovir and trimethoprim-sulfamethoxazole, can worsen leukopenia and thrombocytopenia, narrowing the therapeutic window for patients with TBD, where rejection, infection, and bone marrow failure are constantly competing. Observational data suggest that recipients with the shortest telomeres are more likely to require granulocyte colony-stimulating factor due to leukopenia, indicating that short telomeres are a functional biomarker of haematological vulnerability in patients receiving standard immunosuppressive therapy.

Phenotype-based screening protocols used in ILD transplant evaluations demonstrate a pragmatic approach: a family history of ILD, unexplained liver disease, cytopenia, or macrocytosis identify a subgroup with a “TBD phenotype,” where leukocyte telomere length testing reveals a TL below the tenth percentile in over half of patients. Expert consensus guidelines for telomere biology disorders recommend integrating telomere length assessment into the diagnostic pathway for patients with early-onset pulmonary fibrosis, cryptogenic liver disease, unexplained cytopenias, and a relevant family history. Genetic testing should follow when telomere length falls below predefined percentiles.

Finally, short telomere syndromes should not be considered an absolute contraindication to solid organ transplantation, but rather an invitation to reassess the definition and management of risk. Current data suggests that, with carefully calibrated immunosuppression, intensified infection prophylaxis, and close haematological monitoring, patients with short telomere syndrome can achieve acceptable outcomes, particularly in experienced centres.

Mella et al. assessed the impact of pre-existing haematological conditions on certain vital signs following kidney transplantation. They analysed the incidence of transplant survival, acute rejection, and long-term clinical outcomes. Pre-existing onco-haematological conditions should no longer be considered an automatic contraindication to kidney transplantation, but rather a high-risk factor that requires rigorous selection and follow-up, rather than exclusion.

30 out of 2871 patients (1.0%) who underwent 31 of 3019 kidney transplants had a documented pre-existing haematological condition, including plasma cell dyscrasias, acute leukaemias, high-grade lymphomas or previous PTLD, MPN/MDS-MPN, and hereditary or AL-type amyloidosis. The median time from haematological remission or stabilisation to transplantation ranged from approximately 3 to 12 years, depending on the disease category, reflecting a deliberately conservative selection strategy. When comparing KTRs without haematological disease, matched for age, sex, dialysis modality, and pre-transplant eGFR, no significant differences were found in graft survival at the time of death, patient survival, acute rejection, or major infectious complications. Haematological relapse occurred in only 2 out of 30 patients (6.7%), both of whom were successfully treated without damage to the allograft.

This confirms that, in highly selected candidates, relapse is not necessarily synonymous with graft failure. Furthermore, both cohorts experienced multiple episodes of infection, with no significant differences observed between the two groups.

This study is necessarily limited by its small sample size, single-centre design, and the absence of routine biopsies to assess transplant damage caused by disease recurrence. Nevertheless, we can draw some important conclusions:

- A pre-existing oncohaematological condition should prompt a more thorough assessment, rather than an automatic exclusion.
- With a well-documented, stable disease, careful multidisciplinary supervision, and structured monitoring protocols, these patients can achieve excellent transplant survival, low rejection rates, and favourable long-term clinical outcomes.
- Specific disease surveillance programmes before and after transplantation, including bone marrow assessment, imaging, and biomarker monitoring (e.g., light chains, JAK2 or BCR-ABL status, minimal residual disease testing) tailored to each condition.
- Immunosuppressive therapies that explicitly consider bone marrow reserve and the risk of relapse, avoiding unnecessary lymphodepletion.

Zheng et al. evaluated the effectiveness of treating acute GVHD following liver transplantation. Graft-versus-host disease (GVHD) is a rare complication following liver transplantation (LT), with an incidence of 0.5-2%. GVHD occurs when the donor’s immune cells recognise the recipient’s antigens as foreign and trigger an immune response. Transplants containing a higher number of immunocompetent donor lymphocytes, such as those involving hematopoietic stem cells, bone marrow, or peripheral blood stem cells, are associated with a high incidence of GVHD. Among solid organ transplants, intestinal transplantation has the highest incidence of GVHD, followed by liver transplantation. GVHD rates are lower after kidney, heart, or pancreas transplants.

Early diagnosis of GVHD is important to reduce the severity of infections and mortality rates. Unfortunately, diagnosis can be delayed, as similar symptoms can be observed in cases of drug reactions and bacterial or viral infections. Diagnosis is based on clinical and histological evidence, as well as the presence of chimerism.

Historically, the standard treatment for post-LTx GVHD has relied on high-dose corticosteroids and calcineurin inhibitors. However, the poor results from the historical cohort highlight the inadequacy of this treatment.

ATG is a polyclonal preparation of IgG directed against multiple T-cell surface antigens, including CD2, CD3, CD4, CD8, HLA-DR, and adhesion and co-stimulatory molecules. It induces complement-dependent lysis and Fc-mediated phagocytosis of T cells, leading to a rapid quantitative depletion of circulating donor T lymphocytes involved in tissue trafficking. It promotes a less alloreactive immune reconstitution by reducing the pool of clones strongly reactive to the donor and facilitating repopulation from naïve or less alloreactive precursors.

The study by Zheng et al. provides encouraging, albeit preliminary, evidence that an anti-thymocyte globulin (ATG) regimen can significantly alter the outcomes of acute GVHD associated with liver transplantation. All three patients treated consecutively achieved complete remission, with a median time from diagnosis to remission of 35 days. No serious infectious complications or transplant rejection were reported during follow-up. Compared to the historical cohort, the ATG group demonstrated a higher rate of complete remission (100% vs. 20%), a lower infection rate (0% vs. 60%), and a shorter median time to remission (35 vs. 62 days). The effect size and exploratory p-values, although derived from a small sample, are surprising.

Although limited by the sample size, these observations are noteworthy for several reasons:

- Firstly, the study highlights the importance of early diagnosis, which integrates compatible clinical features, histological confirmation, and analysis of donor T-cell chimerism – a triad that can distinguish aGVHD from mimics.
- Secondly, it leverages ATG’s ability to deplete the donor’s activated lymphocytes, potentially restoring immune balance with less collateral damage than prolonged high-dose corticosteroid therapy.

The inclusion of ruxolitinib, a JAK1/2 inhibitor with proven efficacy in steroid-refractory aGVHD following hematopoietic transplantation, is another rational element. Case reports and small series have already suggested a role for ruxolitinib in GVHD following solid organ transplants, including liver transplants, although experience remains limited. By blocking JAK-STAT signalling downstream of multiple inflammatory cytokines, ruxolitinib can interrupt the “cytokine storm” that perpetuates tissue damage,.

Tai et al. evaluated the influence of immunosuppression on the onset of haematological complications following SOT. They considered different types of immunosuppressive regimens applied after kidney transplantation.

The era of “one-size-fits-all” maintenance immunosuppression in kidney transplantation is clearly ending, yet our routine tools to guide drug choice and intensity are still represented by creatinine, trough levels, DSA, and perhaps protocol biopsies.

That said, the comprehensive characterization of peripheral immune cells in kidney transplant recipients (KTRs), including myeloid−derived suppressor cells (MDSCs), T−cell subsets, and HLA−DR+ monocytes following distinct immunosuppressive treatments, is more than just a descriptive academic exercise: it provide a model for functional profiling of patient immunosuppression.

The paper by Tai et al. offers one of the most comprehensive snapshots to date of circulating immune cells in kidney transplant recipients (KTRs) under different maintenance regimens, including characterization of myeloid-derived suppressor cells (MDSCs) alongside conventional lymphocyte subsets and monocytes.

They suggests that certain peripheral patterns—such as elevated MDSCs with attenuated activated T cells and reduced HLA-DR+ monocytes—may correspond to a more profoundly suppressed state, with implications for infection risk.

This study utilises multiparametric flow cytometry in a real-world cohort of 26 KTRs approximately one year post-transplant and 13 healthy controls, comparing sirolimus (SRL/mTOR inhibitor)–based and tacrolimus (TAC)–based regimens. The investigators show a striking divergence between “clinical stability” and immune homeostasis. In KTRs, granulocytic and monocytic MDSCs (G-MDSCs and M-MDSCs) are markedly increased compared with healthy controls, whereas HLA-DR+ monocytes, activated CD38+/CD28+ T cells, and both naïve and memory CD4+ T-cell subsets are significantly depleted.

The article by Tai et al. is therefore valuable for two reasons:

- Firstly, it recognises that peripheral blood contains a rich, untapped archive of how different immunosuppressive treatments reprogram the immune system of KTRs; and.
- Secondly, it insists that future clinical studies on treatment regimens integrate standardised, high-content peripheral immune monitoring as the primary endpoint, rather than as an additional exploratory component.

Only by systematically linking these cellular signatures to rejection, infection, malignancy, and transplant longevity will we be able to move from descriptive immunophenotyping to truly mechanistic and patient-specific immunosuppression in kidney transplantation.

*In conclusion, b*lood disorders following organ transplantation remain a concerning condition, despite improved understanding of risk factors and their pathophysiology. Indeed, their treatment remains complex due to the wide variety of clinical forms and their progression. Treatment needs to be tailored to each situation. Furthermore, patients undergoing organ transplantation with a haematological condition are more vulnerable, and cytotoxic drugs should be used with caution.

